# Recommendations for Diagnosing and Quantifying treatment outcomes in clinical trials of compulsive sexual behavior disorder^☆^

**DOI:** 10.1016/j.abrep.2025.100610

**Published:** 2025-04-17

**Authors:** Jannis Engel, Shane W. Kraus, Li Yan McCurdy, Marc N. Potenza

**Affiliations:** a Department of Psychiatry, Social Psychiatry and Psychotherapy, Hannover Medical School, Hannover, Germany; b Department of Psychology, University of Nevada Las Vegas, Las Vegas, NV, USA; c Department of Psychiatry, Yale University School of Medicine, New Haven, CT, USA; d Departments of Neuroscience and the Child Study Center, Yale University School of Medicine, New Haven, CT, USA; e Connecticut Council on Problem Gambling, Wethersfield, CT, USA; f Connecticut Mental Health Center, New Haven, CT, USA; g Wu Tsai Institute, Yale University, New Haven, CT, USA

**Keywords:** Compulsive sexual behavior disorder, Problematic pornography consumption, Quantifying treatment outcomes

## Abstract

This article proposes minimum requirements for reporting efficacy in treatment studies of compulsive sexual behavior (CSB). CSB disorder (CSBD) is a condition whose diagnostic criteria were only recently defined by the World Health Organization. Multiple primary and secondary outcomes have been used in treatment trials of CSB, and possible neuropsychological measures have been considered. We suggest including clinical interviews, specifying the nature of the problem behavior and ensuring that the concerns are not entirely related to distress linked to moral judgments or paraphilic interests exclusively. The minimum requirements of reporting the efficacy of behavioral and pharmaceutical treatment outcome studies proposed are measures of: *CSB symptom severity* – problems in personal health, relationships, work and finance; these measures may be complemented by additional measures of quality of life or sexual health; *behavioral engagement in CSB −* frequency (in days per week) of CSB, including time spent thinking about or engaged in the pursuit of CSB*; change processes* – assessing mechanisms of change hypothesized. There are currently no objective (i.e., neuropsychological) measures that can provide a valid picture of the success of therapies. However, promising areas in the brain which reflect treatment changes include prefrontal areas and the reward system. We believe that the guidelines presented should promote harmonized clinical research involving the treatment of CSB and CSBD.

## Highlights

• Minimum requirements for reporting efficacy in CSB treatment studies are needed.
• Clinical interviews are recommended to ensure problems are not solely linked to moral judgments or paraphilic interests.
• Suggestion of reporting: symptom severity, behavioral engagement (frequency/time), and mechanisms of change.
• No objective neuropsychological measures yet exist, but the prefrontal cortex and reward system may index treatment response.

## 1 Introduction

In the field of compulsive sexual behavior (CSB) and CSB disorder (CSBD), more rigorous and systematic methodological approaches are needed for studies of clinical interventions (Antons et al., 2022). Although discussions regarding definitions and classifications of hypersexual disorder, sexual addiction and problematic pornography use (PPU) are fruitful, CSBD in the eleventh revision of the International Classification of Diseases (ICD-11) includes a widely agreed-upon diagnostic entity (WHO, 2019). Thus, intervention studies will likely increase in the near future. In this article, we propose minimal elements for the conduct of intervention studies investigating effective treatments for CSB and CSBD, following upon prior efforts generally in the field of behavioral addictions (Walker et al., 2006) and for pharmacological trials aiming to help people with CSBD (Turner et al., 2022).

To date, multiple outcomes have been employed to assess change in treatment studies of non-paraphilic, dysregulated sexual behavior. For example, self-reported symptom severity (Hallberg, Kaldo, Arver, & Dhejne, 2019) or behavioral engagement (Crosby & Twohig, 2016) have been primary outcomes, and no study to date has used objective (e.g., neuroimaging) measures. However, measures of symptom severity and of behavioral engagement have differed considerably across studies. While this can be seen as appropriate, as variations in treatments may be reflected in different study aims, it may also complicate cross-study comparisons.

In the present article, we propose minimum requirements for reporting on and measuring efficacy in treatment studies. The proposed requirements are intended not to be restrictive, but rather to promote harmonization that will help cross-study comparisons, thus benefiting treatment development efforts for CSB and CSBD.

## 2 Proposed standardized characterization of CSB

### 2.1 Diagnosis of compulsive sexual behavior disorder

Despite the ongoing debate about the etiological classification of CSB, we recommend using the ICD-11 criteria for CSBD as a basis for standardization across studies examining non-paraphilic, dysregulated (“out-of-control”) sexual behaviors. If investigators wish to examine additional criteria that are not included in the ICD-11 criteria, we recommend considering additional outcome factors within a structured framework. For example, to assess emotional regulation, listed as an additional clinical feature in the diagnosis of CSBD (Briken et al., 2024; WHO, 2019), one could consider additionally assessing criteria for hypersexual disorder, which overlap with some but not all diagnostic criteria (Kafka, 2010, Lew-Starowicz et al., 2020). Alternatively, if improving emotional regulation were a specified therapeutic goal, including measures that investigate mechanisms of treatment change may also be warranted. Another example involves the consideration of CSBD as a behavioral addiction. Since some have proposed CSB as a behavioral addiction (Brand et al., 2016, Brand et al., 2019, Brand et al., 2025a, Brand et al., 2025b), including within theoretical frameworks like the Interaction of Person, Affect, Cognition and Execution (I-PACE) model, it may be helpful to include related constructs such as the core features of addictions (e.g., impaired control, increasing priority and continued or escalating engagement despite adverse consequences, or related constructs like craving) or the presence of other behavioral addictions (WHO, 2019).

As concerns were recently raised by a scientific committee of the International Society for Sexual Medicine regarding the pathologization of healthy sexual behavior (Briken et al., 2024), we recommend that the study samples should be carefully assessed for CSB. For example, distress related solely to moral judgments and disapproval of sexual impulses, urges or behaviors is not sufficient to make a diagnosis of CSBD (WHO, 2019), with moral incongruence implicated in other behavioral addictions although not defined in their diagnostic criteria (Lewczuk et al., 2021). Clinical interviewers should be particularly familiar with the field of paraphilic disorders, as these are listed as an exclusion criterion for CSBD. However, paraphilic interests are prevalent in men with CSB and frequently co-occur with CSBD (Engel et al., 2024a). We propose that it is important to assess in a clinical interview the primary source of distress and whether it relates to paraphilic concerns or CSB, for example. Moreover, in the context of sexuality, clinicians and researchers should attentively keep in mind distinguishing between their values and the patient's treatment concerns (Althof, 2012, Briken et al., 2024). Generally, we advise against using questionnaires alone for diagnosis, as establishing diagnoses are best achieved through clinical assessment conducted by experienced clinical interviewers with sexual therapy expertise. For example, multiple factors may influence responses to questionnaires, including understanding of questions and individual respondent characteristics relating to insight, shame, denial/ambivalence, stigma and other factors. Clinicians may incorporate judgment based on clinical contexts and their experience to inform their assessments based on diagnostic criteria.

### 2.2 Description of behavior

CSB can include multiple, non-mutually exclusive behaviors, and patients often show more than one concern. Accordingly, patients report different foci of behaviors that may change over time. Frequently reported domains often rated as out of control (Kafka, 2010, Reid et al., 2012) include:

• Problematic pornography consumption;
• (Online) dating;
• Excessive masturbation;
• Sexual behavior with consenting adults;
• Paid services (webcam sex, telephone sex, prostitution, strip clubs).

Behaviors performed by patients should be recorded, at the onset and conclusion of treatment and at intermittent stages. Although the increased access, affordability and perceived anonymity regarding sexual behaviors (Cooper, 1998) has been questioned (Byers, Menyies, & O’Grady, 2004), the roles of digital technologies continue to evolve. As such, we recommend accurately describing the extent to which behaviors are performed online or offline, in line with ICD-11 recommendations for disorders due to addictive behaviors (WHO, 2019).

## 3 Proposed primary outcome variables

Due to the diversity of behaviors subsumed under CSB, a primary outcome variable alone may not adequately reflect treatment success. Nonetheless, the number of outcome variables should be minimized to reduce multiple comparisons. As a minimum requirement, we recommend collecting measures of symptom severity as a meaningful psychological factor and combining this with additional measures of behavioral engagement.

### 3.1 Symptom severity

Self-report measures of symptom severity help to capture the perceived consequences of CSB for the individual. Basically, together with measures of behavioral engagement, they form a more complete picture than one measure alone. Diagnostically, these measures help to distinguish whether sexual behavior may be best categorized as compulsive. For example, it should be explored more closely if there is a high perceived symptom severity with low behavioral engagement. In these cases, there may be an increased likelihood that sexual behavior is perceived as problematic by those affected due to moral judgments. In addition to self-report measures, we suggest the inclusion of clinical interviews to assess CSBD-related symptom severity. CSBD assessments may be categorical (diagnosis yes/no) or dimensional (numbers of symptom present at assessment).

Several measures of CSB symptom severity have been reported, with varying levels of reliability and validity (Montgomery-Graham, 2017). We propose utilizing measures that are derived from the ICD-11 diagnostic criteria for CSBD, such as the established CSBD-19 (Bőthe et al., 2020) or the recently published 7-item CSBD diagnostic inventory, which appears promising (CSBD-DI, Grubbs et al., 2023).

Concurrent validity has been assessed in some studies of CSB. For example, agreement between clinician-derived diagnoses of hypersexual disorder and data from questionnaires have been examined with respect to inter-rater reliability (Reid et al., 2012). An under-explored area in CSB treatment involves validation via collateral reports (e.g., from relatives of the affected person), which could provide information regarding potential effects of the disorder on social relationships. In principle, these can be a valuable addition, since interpersonal difficulties are prevalent in CSB (Engel et al., 2023a) but the clinical benefit in the area of CSB is still uncertain, as some forms of CSB, like problematic pornography consumption (PPU), can be conducted surreptitiously.

### 3.2 Behavioral engagement

Currently, relationships are incompletely understood between CSB severity and different indices of involvement measured by time or frequency. In fact, there are inconsistent associations between measures of total sexual outlet and CSB (Kingston et al., 2020); similar inconsistencies exist with PPU (Chen et al., 2022). However, we propose that measures of behavioral engagement should be included to provide a comprehensive picture of problematic behaviors that may involve frequency or duration or sexual outlets. Consideration of types of CSB (e.g., PPU, dating behaviors or excessive masturbation) may be relevant. For example, frequency of number of days of pornography consumptions per week and duration of viewing can be measured by time per week or in the form of thinking about sexual behaviors, keeping in mind that there may be correlations between these measures. However, clinical examples may show considerable variance in the behaviors. For example, some patients spend very little time consuming pornography (in each session) while at the same time engaging in the behavior at a high frequency. Other patients, however, exhibit pornography-binging behavior, which often involves watching pornography for long periods, but without necessarily exhibiting this behavior at a high frequency (Wordecha et al., 2018). Therefore, specifying frequency separately from time is recommended.

The time that those affected spend engaged in CSB may be difficult to measure and therefore incompletely understood. More accurate measurements may be derived from recent assessments (e.g., over the past week). However, since there may be effects related to the week before the survey (for example, related to potential shame patients may experience when reporting their behaviors to a clinician), it may be beneficial to additionally report other behavioral engagement (e.g., in a typical week of the month). Because of the scarcity of studies on the reliability of measurements of CSB, it may make sense to draw on experiences of problem gambling, which suggest that the reliability of the reported frequency of days on which gambling occurred is acceptable, while the time spent may be more difficult to recall (Hodgins & Makarchuk, 2003). Thus, our recommendation is to assess the frequency of CSB in days per week in the week prior or in a typical week of the last month.

If study participants indicate abstinence or a reduction in problematic behaviors, additional information regarding how the behaviors may remain concerning (e.g., through preoccupation) should be assessed as appropriate. Preoccupation with sexual content may extend beyond actively engaging in the problematic behavior and may include craving or urges (Way & Kraus, 2024). The degree of preoccupation may depend upon the type of CSB. For example, engaging in PPU may take more time because of the availability of online explicit materials than going out on dates with a limited number of partners. In addition, it is difficult to determine the time of sexual thoughts that many patients report when they are exposed to sexual cues, such as seeing an attractive person. Moreover, thoughts related to CSB may also include thinking about possible adverse consequences of the behavior, such as the possible loss of a job or relationship. Preoccupation can therefore include either cognitive time spent thinking about the problematic behavior or its consequences. Despite such complexities, mental preoccupation remains an important measure in considering the severity of CSB, as in other behavioral addictions (Kim et al., 2009).

## 4 Proposed secondary outcome variables

While the proposed primary outcome measures can capture elements of treatment efficacy, other variables should also be collected to contextualize the results. For example, reduced frequency of CSB may be associated with higher life satisfaction if a sex-positive intervention reinforces the notion that a fulfilling sexual life is a fundamental human need and increases satisfaction in one’s sexual life (Coleman et al., 2018). However, reduced frequency of CSB may instead by associated with lower life satisfaction if it entails being socially isolated in order to achieve abstinence. The negative impact of CSB on other areas of functioning parallels that of other behavioral addictions, such as gambling disorder (Dickerson, 1989, Walker et al., 2006):

• Mental health;
• Relationships, marital and family;
• Financial;
• Employment and productivity; and
• Legal problems/offenses.

Although there are now many instruments that cover the above areas, few have been clinically validated for CSB, especially in larger samples that have only been studied online. In the intervention studies to date, some of the other relevant issues have been recorded as secondary outcomes, although there is considerable variability in the instruments used (Antons et al., 2022). Given the scarce data currently available to clinicians, we do not make specific recommendations regarding which measures are most recommended to assess other areas of functioning.

## 5 Potential mediators/moderators and biomarkers of treatment efficacy

In many interventions (e.g., psychotherapies), common factors such as therapeutic alliance may influence the success or failure of an intervention (Wampold, 2015). For CSB, the ratio of the magnitude of influence between specific vs. non-specific influencing factors is not sufficiently understood. However, the assumption that specific factors may have a causal influence on treatment success is relevant for the selection of interventions. Despite the general presence of non-specific factors, we recommend reporting which intervention addresses which domain of CSB. However, the effects of specific interventions may not be limited to the addressed behavior but may also influence other (problematic) behaviors.

In substance use disorders (SUDs) such as cocaine use disorder, there are clinically significant endpoints (e.g., Loya et al., 2023) that are not currently present in CSB due to limited information regarding relevant somatic markers. One possibility for objectively mapping treatment success lies in neuroimaging, for which initial findings are available, particularly in SUDs and gaming and gambling disorders (Mestre-Bach & Potenza, 2023). Imaging-derived measures may include, for example, task-based activations in the brain, which have shown promise in previous studies for mapping treatment success (DeVito et al., 2019, Balodis et al., 2016, Zhang et al., 2016;). Moreover, measurements at the onset of treatment may predict treatment success (Huhn et al., 2019, Potenza et al., 2013, Xu et al., 2010, Xu et al., 2014). Although these possibilities are promising and reviews have shown that neurobiological and neuropsychological alterations are present in affected men with CSB (e.g., Engel et al., 2024b, Stark, Klucken, Potenza, Brand, & Strahler, 2018), there are currently no valid neurobiological measures available to map treatment success in CSB. The neurobiological changes in individuals with CSB likely differ from those without CSB to varying degrees, depending on the progress of the disorder. In the case of CSB, changes in structure or changes in brain activity in the area of the dorsal striatum and dorsolateral prefrontal cortex have been hypothesized (Brand, 2022) or the superior frontal lobe (Engel et al., 2023b). Other potential areas that may show changes include areas of the reward system, e.g., ventral tegmental area, ventral striatum, ventromedial prefrontal cortex (Brand, 2022, Gola et al., 2017). Considering brain networks in this regard is also important (Antons et al., 2023, Antons et al., 2024, Lichenstein et al., 2021, Yip et al., 2019). These and other examples may be considered and may differ based on the anticipated active ingredients of the therapy.

## 6 Other variables of interest

As therapy adherence is an important outcome factor, it is necessary to report treatment attrition (Caetano, 2004). We suggest researchers use a CONSORT flow diagram, monitoring the number of individuals at each state of the treatment program (Falci & Marques, 2015). Reporting on treatment attrition in CSB should consider the following:

• Total number of individuals seeking treatment;
• Number of patients defined by intention-to-treat;
• Number of patients entering treatment (at least one treatment session);
• Number of patients completing treatment; and
• Number of patients in each category receiving follow-up evaluations.

While it has been emphasized in problem gambling that treatment tolerance is particularly important in pharmacological studies (Walker et al., 2006), we would like to extend this recommendation more clearly to all interventions in CSB. For pharmacological treatments, we suggest the inclusion of Dosage Record and Emergent Symptom Scale (DOTES; Guy, 1976) for assessing the emergence of adverse effects. To assess efficacy and effectiveness, statistical analyses should include both intention-to-treat and completer analyses (Walker et al., 2006).

Since some pharmacological treatments (e.g., selective serotonin uptake inhibitors (SSRIs)) may have an influence on sexuality that goes beyond problematic behavior, we recommend including measures of sexual dysfunction and partnered sexuality. For example, SSRIs may adversely impact sexuality (Marks, 2023), as well as reduce premature ejaculation (Saleh, Majzoub, & Abu El-Hamd, 2021). With these considerations, multiple sexual measures should be assessed.

## 7 Assessment points

Often interventions are of short durations and therefore may not cover time periods for all relevant changes to occur. For example, relational tensions related to problematic behavior may not immediately disappear as a result of the intervention, even if a significant reduction in CSB has occurred by the end of the intervention. Specifically, partnership problems may not be resolved after the intervention has formally ended. Additionally, legal problems that occur (albeit rarely) also require processing that often exceeds the duration of the intervention. Therefore, we recommend the inclusion of follow-up periods in order to adequately assess longer-term effects of the therapy. Useful points of assessment are:

• Commencement of therapy;
• Post-treatment;
• Short-term follow-up (3–6 months following completion of treatment), and;
• Medium term (one year following completion of treatment).

We acknowledge, however, that conducting longer-term follow-up measurements (i.e., 12 months or greater) may be particularly difficult from multiple perspectives. In addition, the longer it has been since an intervention was conducted, the higher the expected drop-out, which may limit validity, particularly in studies that may have been powered for shorter-term treatment.

## 8 Summary

Following the approach proposed here can help to standardize the reporting of interventions in CSB and CSBD. We would like to emphasize the following points:

• The sample should be accurately described in terms of which CSB is specifically present and how the clinical validation of CSB was ensured, especially in the case of CSBD.
• Instruments selected for screening and assessment should have empirical support. For example, the CSBD-19 maps onto ICD-11 criteria for CSBD (Bőthe et al., 2020) and has considerable empirical support, although a shorter version (the CSBD-DI, Grubbs et al., 2023) may have merit as more data are accumulated
• Outcome variables should include measures of clinical impairment and behavioral engagement in frequency or duration.
• Adverse events (especially in relation to sexual functioning) should be assessed, as should secondary outcomes such as other relevant areas of functioning.
• At present, there are no objective (neurophysiological) measures that may accurately reflect treatment success. However, there are potentially relevant brain regions and circuitry that may relate importantly to treatment effects.
• Treatment attrition should be described precisely and measures (e.g., intention-to-treat) should be included in the analyses.

## CRediT authorship contribution statement

**Jannis Engel:** Writing – original draft, Conceptualization. **Shane W. Kraus:** Writing – review & editing, Supervision, Conceptualization. **Li Yan McCurdy:** Writing – original draft, Conceptualization. **Marc N. Potenza:** Writing – review & editing, Supervision, Conceptualization.

## Declaration of competing interest

The authors declare the following financial interests/personal relationships which may be considered as potential competing interests: Marc Potenza reports a relationship with Mohegan Sun that includes: funding grants. Marc Potenza reports a relationship with Boehringer Ingelheim GmbH that includes: consulting or advisory. Marc Potenza has patent Glutamate pending to Novartis and Yale. The authors have no conflicts of interest to report. Marc Potenza discloses the following. MNP has consulted for Baria-Tek and Boehringer Ingelheim; has been involved in a patent application with Yale University and Novartis; has received research support from Mohegan Sun Casino and the Connecticut Council on Problem Gambling; has participated in surveys, mailings or telephone consultations related to drug addiction, internet use, impulse-control disorders or other health topics; has consulted for and/or advised gambling, non-profit, healthcare and legal entities on issues related to internet use, impulse control and addictive disorders; has performed grant reviews for research-funding agencies; has edited journals and journal sections including being on the editorial board of Addictive Behaviors Reports; has given academic lectures in grand rounds, CME events and other clinical or scientific venues; and has generated books or book chapters for publishers of mental health texts. If there are other authors, they declare that they have no known competing financial interests or personal relationships that could have appeared to influence the work reported in this paper.

## Data Availability

No data was used for the research described in the article.
